# Supplementary material for: Perinatal intimate partner violence and breastfeeding practices: A systematic review and meta-analysis protocol
Source: PLoS One. 2025 Feb 6;20(2):e0318585. doi: 10.1371/journal.pone.0318585 (PMC11801569; doi:10.1371/journal.pone.0318585)
Supplement: S2 File — (DOCX) [file pone.0318585.s002.docx]

**Appendix, S2 File. Search strategies for included databases**

**Medline search: searched on the 17^th^ of June with a total result of 917 articles**

AB ( Breastfeeding OR “breast feeding” OR (breast milk expressio*) OR (milk collectio*) OR (feeding behaviou*) OR (feeding patter*) OR (feeding practic*) OR (lactatio*) OR “milk ejection” OR nursing OR “infant feeding” OR “exclusive breastfeeding” OR “breastfeeding duration” OR “breastfeeding intention” OR “pumping breast” OR “human milk” OR “breast milk” OR (milk secretio*) OR “milk let down” OR “exclusive breast feeding” OR “exclusive breast feedings” OR “breast feedings” OR “breast pumping” ) AND AB ( "intimate partner violence” OR "domestic violence" OR “domestic abuse” OR "partner abuse” OR “spouse abuse” OR “spousal abuse” OR “spousal violence” OR battering OR “battered women” OR “battered woman” OR "family violence" OR "relationship violence” OR “relationship aggression” OR “domestic abuse” OR “partner violence” OR “physical abuse” OR “physical violence” OR “physical maltreatment” OR “dating violence” OR “partner homicide” OR “psychological abuse” OR “wife abuse” OR “sex offences” OR “sexual violence” OR “sexual harm” OR “sexual coercion” OR “abused woman” OR “abused women” OR “couple violence” OR “wife beating” OR “physical harm” OR “physical aggression” OR “emotional violence” OR “emotional abuse” OR “emotional harm” OR “violence against women” )

**PubMed search: searched on the 18^th^ of June with a total result of 1158 articles**

(Breastfeeding[Title/Abstract] OR "breast feeding"[Title/Abstract] OR (breast milk expressio*[Title/Abstract]) OR (milk collectio*[Title/Abstract]) OR (feeding behaviou*[Title/Abstract]) OR (feeding patter*[Title/Abstract]) OR (feeding practic*[Title/Abstract]) OR (lactatio*[Title/Abstract]) OR "milk ejection"[Title/Abstract] OR nursing[Title/Abstract] OR "infant feeding"[Title/Abstract] OR "exclusive breastfeeding"[Title/Abstract] OR "breastfeeding duration"[Title/Abstract] OR "breastfeeding intention"[Title/Abstract] OR "pumping breast"[Title/Abstract] OR "human milk"[Title/Abstract] OR "breast milk"[Title/Abstract] OR (milk secretio*[Title/Abstract]) OR "milk let down"[Title/Abstract] OR "exclusive breast feeding"[Title/Abstract] OR "exclusive breast feedings"[Title/Abstract] OR "breast feedings"[Title/Abstract] OR "breast pumping"[Title/Abstract]) AND ("intimate partner violence"[Title/Abstract] OR "domestic violence"[Title/Abstract] OR "domestic abuse"[Title/Abstract] OR "partner abuse"[Title/Abstract] OR "spouse abuse"[Title/Abstract] OR "spousal abuse"[Title/Abstract] OR "spousal violence"[Title/Abstract] OR battering[Title/Abstract] OR "battered women"[Title/Abstract] OR "battered woman"[Title/Abstract] OR "family violence"[Title/Abstract] OR "relationship violence"[Title/Abstract] OR "relationship aggression"[Title/Abstract] OR "domestic abuse"[Title/Abstract] OR "partner violence"[Title/Abstract] OR "physical abuse"[Title/Abstract] OR "physical violence"[Title/Abstract] OR "physical maltreatment"[Title/Abstract] OR "dating violence"[Title/Abstract] OR "partner homicide"[Title/Abstract] OR "psychological abuse"[Title/Abstract] OR "wife abuse"[Title/Abstract] OR "sex offences"[Title/Abstract] OR "sexual violence"[Title/Abstract] OR "sexual harm"[Title/Abstract] OR "sexual coercion"[Title/Abstract] OR "abused woman"[Title/Abstract] OR "abused women"[Title/Abstract] OR "couple violence"[Title/Abstract] OR "wife beating"[Title/Abstract] OR "physical harm"[Title/Abstract] OR "physical aggression"[Title/Abstract] OR "emotional violence"[Title/Abstract] OR "emotional abuse"[Title/Abstract] OR "emotional harm"[Title/Abstract] OR "violence against women"[Title/Abstract])

**Scopus search: searched on the 19^th^ of June with a total result of 651 articles**

TITLE-ABS-KEY ("breast feeding") OR TITLE-ABS-KEY ("breastfeeding intention") OR TITLE-ABS-KEY ("breastfeeding") OR TITLE-ABS-KEY ("breastfeeding duration") OR TITLE-ABS-KEY ("exclusive breast feeding") OR TITLE-ABS-KEY ("exclusive breastfeeding") OR TITLE-ABS-KEY ("breast milk expressio*") OR TITLE-ABS-KEY ("breastmilk expressio*") OR TITLE-ABS-KEY ("milk collectio*") OR TITLE-ABS-KEY ("breast pumping") OR TITLE-ABS-KEY ("pumping breast") OR TITLE-ABS-KEY ("feeding behavior") OR TITLE-ABS-KEY ("feeding patter*") OR TITLE-ABS-KEY ("human milk") OR TITLE-ABS-KEY ("milk, human") OR TITLE-ABS-KEY ("breast milk") OR TITLE-ABS-KEY("lactatio*") OR TITLE-ABS-KEY ("milk secretion") OR TITLE-ABS-KEY ("milk ejection") OR TITLE-ABS-KEY ("milk let-down") AND TITLE-ABS-KEY ("intimate partner violence") OR TITLE-ABS-KEY ("dating violence") OR TITLE-ABS-KEY ("partner violence") OR TITLE-ABS-KEY ("partner violence") OR TITLE-ABS-KEY ("partner homicide") OR TITLE-ABS-KEY ("psychological violence") OR TITLE-ABS-KEY ("psychological abuse") OR TITLE-ABS-KEY ("spous* abuse") OR TITLE-ABS-KEY ("wife abuse") OR TITLE-ABS-KEY ("partner abuse") OR TITLE-ABS-KEY ("domestic violence") OR TITLE-ABS-KEY ("family violence") OR TITLE-ABS-KEY ("physical abuse") OR TITLE-ABS-KEY ("physical violence") OR TITLE-ABS-KEY ("physical maltreatment") OR TITLE-ABS-KEY ("sex offenses") OR TITLE-ABS-KEY ("sexual violence") OR TITLE-ABS-KEY ("sexual abuse") OR TITLE-ABS-KEY ("sexual harm") OR TITLE-ABS-KEY ("sexual coercion") OR TITLE-ABS-KEY ("battered wom?n") OR TITLE-ABS-KEY ("abused wom?n") OR TITLE-ABS-KEY ("relationship aggression") OR TITLE-ABS-KEY ("couple violence") OR TITLE-ABS-KEY ("relationship violence") OR TITLE-ABS-KEY ("spousal violence") OR TITLE-ABS-KEY ("domestic violence") OR TITLE-ABS-KEY ("wife beating") OR TITLE-ABS-KEY ("physical harm") OR TITLE-ABS-KEY ("physical aggression") OR TITLE-ABS-KEY ("emotional violence") OR TITLE-ABS-KEY ("emotional abuse") OR TITLE-ABS-KEY ("emotional harm") OR TITLE-ABS-KEY ("controlling behavior") OR TITLE-ABS-KEY ("violence against women")

**Web of science search: searched on the 17^th^ of June with a total result of 3031 articles**

(TS=("intimate partner violence” OR "domestic violence" OR “domestic abuse” OR "partner abuse” OR “spouse abuse” OR “spousal abuse” OR “spousal violence” OR battering OR “battered women” OR “battered woman” OR "family violence" OR "relationship violence” OR “relationship aggression” OR “domestic abuse” OR “partner violence” OR “physical abuse” OR “physical violence” OR “physical maltreatment” OR “dating violence” OR “partner homicide” OR “psychological abuse” OR “wife abuse” OR “sex offences” OR “sexual violence” OR “sexual harm” OR “sexual coercion” OR “abused woman” OR “abused women” OR “couple violence” OR “wife beating” OR “physical harm” OR “physical aggression” OR “emotional violence” OR “emotional abuse” OR “emotional harm” OR “violence against women”)) AND TS=(Breastfeeding OR “breast feeding” OR (breast milk expressio*) OR (milk collectio*) OR (feeding behaviou*) OR (feeding patter*) OR (feeding practic*) OR (lactatio*) OR “milk ejection” OR nursing OR “infant feeding” OR “exclusive breastfeeding” OR “breastfeeding duration” OR “breastfeeding intention” OR “pumping breast” OR “human milk” OR “breast milk” OR (milk secretio*) OR “milk let down” OR “exclusive breast feeding” OR “exclusive breast feedings” OR “breast feedings” OR “breast pumping”)

**Informit search: searched on the 17^th^ of June with a total result of 346 articles**

[All Fields:Breastfeeding OR All Fields:“breast feeding” OR All Fields:(breast milk expressio*) OR All Fields:(milk collection*) OR All Fields:(feeding behaviour*) OR All Fields:(feeding patter*) OR All Fields:(feeding practice*) OR All Fields:(lactation*) OR All Fields:“milk ejection” OR All Fields:nursing OR All Fields:“infant feeding” OR All Fields:“exclusive breastfeeding” OR All Fields:“breastfeeding duration” OR All Fields:“breastfeeding intention” OR All Fields:“pumping breast” OR All Fields:“human milk” OR All Fields:“breast milk” OR All Fields:(milk secretio*) OR All Fields:“milk let down” OR All Fields:“exclusive breast feeding” OR All Fields:“exclusive breast feedings” OR All Fields:“breast feedings” OR All Fields:“breast pumping”] AND [All Fields:"intimate partner violence” OR All Fields:"domestic violence" OR All Fields:“domestic abuse” OR All Fields:"partner abuse” OR All Fields:“spouse abuse” OR All Fields:“spousal abuse” OR All Fields:“spousal violence” OR All Fields:battering OR All Fields:“battered women” OR All Fields:“battered woman” OR All Fields:"family violence" OR All Fields:"relationship violence” OR All Fields:“relationship aggression” OR All Fields:“domestic abuse” OR All Fields:“partner violence” OR All Fields:“physical abuse” OR All Fields:“physical violence” OR All Fields:“physical maltreatment” OR All Fields:“dating violence” OR All Fields:“partner homicide” OR All Fields:“psychological abuse” OR All Fields:“wife abuse” OR All Fields:“sex offences” OR All Fields:“sexual violence” OR All Fields:“sexual harm” OR All Fields:“sexual coercion” OR All Fields:“abused woman” OR All Fields:“abused women” OR All Fields:“couple violence” OR All Fields:“wife beating” OR All Fields:“physical harm” OR All Fields:“physical aggression” OR All Fields:“emotional violence” OR All Fields:“emotional abuse” OR All Fields:“emotional harm” OR All Fields:“violence against women”]

**PyscInfo search: searched on the 19^th^ of June with a total result of 681 articles**

Abstract: "intimate partner violence” OR "domestic violence" OR “domestic abuse” OR "partner abuse” OR “spouse abuse” OR “spousal abuse” OR “spousal violence” OR battering OR “battered women” OR “battered woman” OR "family violence" OR "relationship violence” OR “relationship aggression” OR “domestic abuse” OR “partner violence” OR “physical abuse” OR “physical violence” OR “physical maltreatment” OR “dating violence” OR “partner homicide” OR “psychological abuse” OR “wife abuse” OR “sex offences” OR “sexual violence” OR “sexual harm” OR “sexual coercion” OR “abused woman” OR “abused women” OR “couple violence” OR “wife beating” OR “physical harm” OR “physical aggression” OR “emotional violence” OR “emotional abuse” OR “emotional harm” OR “violence against women” AND Abstract: Breastfeeding OR “breast feeding” OR (breast milk expressio*) OR (milk collectio*) OR (feeding behaviou*) OR (feeding patter*) OR (feeding practic*) OR (lactatio*) OR “milk ejection” OR nursing OR “infant feeding” OR “exclusive breastfeeding” OR “breastfeeding duration” OR “breastfeeding intention” OR “pumping breast” OR “human milk” OR “breast milk” OR (milk secretio*) OR “milk let down” OR “exclusive breast feeding” OR “exclusive breast feedings” OR “breast feedings” OR “breast pumping”

**Cochrane library search: searched on the 18^th^ of June with a total result of 139 articles**

Breastfeeding OR “breast feeding” OR (breast milk expressio*) OR (milk collectio*) OR (feeding behaviou*) OR (feeding patter*) OR (feeding practic*) OR (lactatio*) OR “milk ejection” OR nursing OR “infant feeding” OR “exclusive breastfeeding” OR “breastfeeding duration” OR “breastfeeding intention” OR “pumping breast” OR “human milk” OR “breast milk” OR (milk secretio*) OR “milk let down” OR “exclusive breast feeding” OR “exclusive breast feedings” OR “breast feedings” OR “breast pumping” in All Text AND "intimate partner violence" OR "domestic violence" OR “domestic abuse” OR "partner abuse” OR “spouse abuse” OR “spousal abuse” OR “spousal violence” OR battering OR “battered women” OR “battered woman” OR "family violence" OR "relationship violence” OR “relationship aggression” OR “domestic abuse” OR “partner violence” OR “physical abuse” OR “physical violence” OR “physical maltreatment” OR “dating violence” OR “partner homicide” OR “psychological abuse” OR “wife abuse” OR “sex offences” OR “sexual violence” OR “sexual harm” OR “sexual coercion” OR “abused woman” OR “abused women” OR “couple violence” OR “wife beating” OR “physical harm” OR “physical aggression” OR “emotional violence” OR “emotional abuse” OR “emotional harm” OR “violence against women” in All Text

**JBI EBP search: searched on the 15^th^ of June with a total result of 87 articles**

| 1. | ("intimate partner violence" or "domestic violence" or domestic abuse or "partner abuse" or "  spous* abuse").mp. [mp=text, heading word, subject area node word, title] |  | | | |
| --- | --- | --- | --- | --- | --- |
| 2. | ("spousal violence" or battering or "battered wom?n" or "family violence" or "relationship violence"  or "relationship aggression" or "domestic abuse" or "partner violence").mp. [mp=text, heading word,  subject area node word, title] |  | | | |
| 3. | 1 or 2 |  | | | |
| 4. | ("physical abuse" or "physical violence" or "physical maltreatment" or "dating violence" or "  partner homicide" or "psychological abuse").mp. [mp=text, heading word, subject area node word,  title] |  | | | |
| 5. | 3 or 4 |  | | | |
| 6. | ("wife abuse" or "sex offences" or "sexual violence" or "sexual harm" or "sexual coercion" or  "abused wom?n" or "couple violence").mp. [mp=text, heading word, subject area node word, title] |  | | | |
| 7. | 5 or 6 |  | | | |
| 8. | ("wife beating" or "physical harm" or "physical aggression" or "emotional violence" or  "emotional abuse" or "emotional harm" or "violence against women").mp.  [mp=text, heading word, subject area node word, title] |  | | | |
| 9. | 7 or 8 |  | | | |
| 10. | ("Breastfeeding" or "breast feeding" or "breast milk expressio*" or "milk collectio*" or  "feeding behaviou*").mp. [mp=text, heading word, subject area node word, title] |  | | | |
| 11. | ("feeding patter*" or "feeding practic*" or "lactatio*" or "milk ejection" or "nursing" or  "infant feeding" or "exclusive breastfeeding" or "breastfeeding duration" or  "breastfeeding intention").mp. [mp=text, heading word, subject area node word, title] |  | | | |
| 12. | 10 or 11 |  | | | |
| 13. | ("pumping breast" or "human milk" or "breast milk" or "milk secretio*" or "milk let down"  or "exclusive breast feeding" or "exclusive breast feedings" or "breast feedings" or  "breast pumping").mp. [mp=text, heading word, subject area node word, title] |  | | | |
| 14. | 12 or 13 |  | | | |
| 15. | 9 and 14 |  |  |  |  |

**CINAHL search: searched on the 15^th^ of June with a total result of 563** **articles**

AB ( Breastfeeding OR “breast feeding” OR (breast milk expressio*) OR (milk collectio*) OR (feeding behaviou*) OR (feeding patter*) OR (feeding practic*) OR (lactatio*) OR “milk ejection” OR nursing OR “infant feeding” OR “exclusive breastfeeding” OR “breastfeeding duration” OR “breastfeeding intention” OR “pumping breast” OR “human milk” OR “breast milk” OR (milk secretio*) OR “milk let down” OR “exclusive breast feeding” OR “exclusive breast feedings” OR “breast feedings” OR “breast pumping” ) AND AB ( "intimate partner violence” OR "domestic violence" OR “domestic abuse” OR "partner abuse” OR “spouse abuse” OR “spousal abuse” OR “spousal violence” OR battering OR “battered women” OR “battered woman” OR "family violence" OR "relationship violence” OR “relationship aggression” OR “domestic abuse” OR “partner violence” OR “physical abuse” OR “physical violence” OR “physical maltreatment” OR “dating violence” OR “partner homicide” OR “psychological abuse” OR “wife abuse” OR “sex offences” OR “sexual violence” OR “sexual harm” OR “sexual coercion” OR “abused woman” OR “abused women” OR “couple violence” OR “wife beating” OR “physical harm” OR “physical aggression” OR “emotional violence” OR “emotional abuse” OR “emotional harm” OR “violence against women” )
