# Supplementary material for: Perinatal intimate partner violence and breastfeeding practices: A systematic review and meta-analysis protocol
Source: PLoS One. 2025 Feb 6;20(2):e0318585. doi: 10.1371/journal.pone.0318585 (PMC11801569; doi:10.1371/journal.pone.0318585)
Supplement: Attachment — Submitted filename: PLOS ONE Review 2024.docx [file pone.0318585.s003.docx]

**Perinatal intimate partner violence and breastfeeding practices: a systematic review and meta-analysis protocol**

Zelalem Nigussie Azene^*1,3^ Catherine MacPhail^1^, Lisa Gaye Smithers^1,2^

*Corresponding author

^1^School of Health and Society, University of Wollongong, Northfields Road, Wollongong, NSW 2522, Australia

^2^School of Public Health, University of Adelaide, Adelaide, SA, Australia

^3^Department of Women’s and Family Health, School of Midwifery, College of Medicine and Health Sciences, University of Gondar, Gondar, Ethiopia

Emails of authors:

ZNA ([zna772@uowmail.edu.au](mailto:zna772@uowmail.edu.au))

CMP ([cmacphai@uow.edu.au](mailto:cmacphai@uow.edu.au))

LGS ([lsmithers@uow.edu.au](mailto:lsmithers@uow.edu.au))

ORCID iDs

ZNA- <https://orcid.org/0000-0002-3511-7562>

CMP- <https://orcid.org/0000-0002-0614-0157>

LGS- <https://orcid.org/0000-0002-6585-7836>

**Abstract**

**Background**

Intimate partner violence increases the risk of detrimental health, behaviors and psychological issues in mothers, affecting infant nutrition and development. However, the potential effects of maternal exposure to intimate partner violence on breastfeeding practices are understudied, and the results of individual studies are inconsistent and conflicting. The aims of this systematic review and meta-analysis are therefore to 1) estimate the prevalence of perinatal intimate partner violence and, 2) examine the relationship between perinatal intimate partner violence and breastfeeding outcomes.

**Methods and analysis**

This systematic review and meta-analysis will investigate the association between perinatal intimate partner violence and breastfeeding outcomes, including early initiation within 1 hour after giving birth, exclusive breastfeeding under six months, and continued breastfeeding at two years or beyond. Comprehensive searches will be conducted in PsycInfo, Scopus, Web of Science, Medline, Cochrane, JBI EBP, CINAHL, Informit, and PubMed electronic databases. Data extraction will be performed independently by two reviewers, with discrepancies resolved by a third reviewer. Statistical analysis will be conducted using STATA/SE version 17, employing random-effects models to calculate pooled effect sizes and assess heterogeneity with I² and Chi-square tests. Subgroup analyses and meta-regression will explore potential sources of heterogeneity.

**Discussion and conclusion**

Evidence suggests that intimate partner violence is linked to poor breastfeeding outcomes. This systematic review and meta-analysis will update, compile, and critically review the evidence of the role of intimate partner violence on breastfeeding outcomes. This systematic review will inform effective strategies and interventions to support breastfeeding among IPV-affected women, thereby enhancing maternal and child health.

**Ethics and dissemination**

As this review and meta-analysis involves secondary analysis of existing data, ethical approval is not required. Findings will be disseminated through peer-reviewed publications and scientific conferences, aiming to inform strategies to support breastfeeding among women affected by intimate partner violence.

**Study registration**

This protocol is registered with the International Prospective Register of Systematic Reviews (PROSPERO), registration number CRD42024555048.

**Keywords:** Intimate partner violence, Domestic violence, Perinatal period, Breastfeeding outcomes, Early initiation of breastfeeding, Exclusive breastfeeding, Continuation of breastfeeding, Systematic review, Meta-analysis

**Introduction**

Intimate Partner Violence (IPV), the most common form of violence against women, is an important global public health concern, posing a substantial risk to the health and well-being of women (1). It is defined as any behavior within a current or former male intimate relationship that causes physical, sexual, psychological harm, including physical aggression, sexual coercion, psychological abuse, and controlling behaviors (2, 3). Globally, nearly one-third (30%) of women who have been in a relationship report experiencing some form of physical and/or sexual violence by their intimate partner at some point in their lifetime (1), with an increased risk of exposure during and after pregnancy (4).

Pregnancy heightens vulnerability to IPV due to changes in physical, emotional, social, and economic demands and needs. Consequently, certain risk factors may become more significant during this time, potentially causing or exacerbating violence (5). Studies have shown that these risk factors extend beyond the pregnancy period, encompassing the timeframe from 12 months preconception to 12 months after childbirth or postpartum, and represent a period of particular vulnerability to violence (6-10). According to the latest World Health Organization (WHO) estimate, globally, over a quarter (27%) of women aged 15–49 years who have ever been married or partnered have experienced physical or sexual violence by an intimate partner at least once in their lifetime (3). An analysis of demographic and health surveys and the International violence against women survey found that IPV prevalence rates during pregnancy range from 2% in Australia, Denmark, Cambodia, and the Philippines to 13.5% in Uganda, with the majority ranging between 4% and 9% (4). However, this is likely to be underreported, as producing accurate relative prevalence estimates for many conditions in women’s health and healthcare, in general, remains challenging due to widespread underreporting (11). This is particularly true for IPV, which is notoriously underreported; true cases are correctly diagnosed only about 25% of the time, with this probability varying across racial groups (12).

Perinatal IPV has been linked to both fatal and non-fatal adverse health outcomes for women and their babies (13). Non-fatal outcomes associated with IPV during pregnancy encompass a range of adverse pregnancy complications, including low birth weight, premature delivery, miscarriage, abortion, antepartum hemorrhage, intrauterine growth restriction, and perinatal death. Additionally, IPV can lead to negative health behaviors such as drug and alcohol use and smoking, as well as adverse psychosomatic outcomes including physical injuries, depression, anxiety, and suicidal tendencies (14-16). Maternal homicide and suicide are the most extreme fatal outcomes of IPV during pregnancy (17-19). Furthermore, IPV is suggested to adversely affect maternal breastfeeding practices particularly, in fragile settings, thereby compromising infant and young child nutrition (20).

Breastmilk is the ideal food for infants, supplying all the energy and nutrients required during the initial months of life. It continues to meet up to half or more of a child's nutritional needs in the latter half of the first year and up to one-third of their needs during the second year (21). Optimal breastfeeding is crucial for both infants and mothers. It helps reduce the incidence of childhood infections in infants, including diarrhea, ear infections, and pneumonia (22). For mothers, breastfeeding helps lower the risk of postpartum hemorrhage and depression, reduces the likelihood of breast cancer, and decreases the incidence of premature mortality due to various infectious diseases (23, 24). The WHO recommends initiating breastfeeding within the first hour after birth, exclusively breastfeeding for 6 months, and continuing breastfeeding for 2 years or more, alongside complementary feeding (25). Despite the numerous benefits of maternal breastfeeding, a significant proportion of children globally are not breastfed according to the guidelines set by WHO and the United Nations Children’s Fund (UNICEF) (26).

Globally, less than half of newborns begin breastfeeding within the first hour after birth and, only 41% of infants under six months are exclusively breastfed, falling short of the 2030 global target of 70%. While more than two-thirds of mothers continue breastfeeding for at least one year, the rate drops to 45% when the child reaches two years of age (27). Although there is extensive evidence on factors influencing breastfeeding practices, psychosocial factors, such as maternal experience of IPV, remain poorly understood (26). A recent analysis of the 2018 Demographic and Health Survey (DHS) in Cameroon found that emotional and sexual violence were linked to a reduced likelihood of early breastfeeding initiation, whereas physical violence showed no significant association. None of the dimensions of IPV were associated with exclusive breastfeeding, even after adjusting for infant, maternal, and household factors (28). Further, in a recent study using data from the DHS of 51 low-income and middle-income countries, distributed by WHO region, 52.9% (27/51) were from Africa, 11.8% (6/51) from the Americas, 7.8% (4/51) from the Eastern Mediterranean, 11.8% (6/51) from Europe, 11.8% (6/51) from South-East Asia, and 3.9% (2/51) from the Western Pacific, found that mothers exposed to IPV were less likely to initiate breastfeeding early and breastfeed exclusively in the first 6 months.

Given the significant public health implications of IPV and breastfeeding, a systematic review and meta-analysis are essential to establish a clear, evidence-based understanding of their relationship. The current literature on maternal IPV exposure and breastfeeding practices is sparse and conflicting, necessitating further investigation. Currently, there are two systematic reviews examining the association between IPV and breastfeeding practices reviewing studies published between 2001 and 2019 (29, 30). The limitations of the first review included the inappropriate use of the Strengthening the Reporting of Observational Studies in Epidemiology (STROBE) guidelines as a quality assessment tool. STROBE is meant to improve the reporting of observational studies (31) rather than assessing their quality.

Furthermore, the review did not discuss confounding factors (30). Neither review conducted a meta-analysis, highlighting a significant gap in the literature. Moreover, additional literature has recently emerged.

Hence, the aims of this review and meta-analysis are to synthesize existing evidence to determine the prevalence of perinatal IPV and elucidate its association with breastfeeding outcomes. Ultimately, it will inform effective strategies and interventions to support breastfeeding among IPV-affected women and suggest IPV prevention measures for perinatal women, thereby enhancing maternal and child health.

**METHODS**

**Protocol registration and reporting**

This protocol is written according to the guidelines set forth by the Preferred Reporting Items for Systematic Reviews and Meta-Analyses (PRISMA) of observational studies in epidemiology guidelines (32) (**S1 File)** and is registered with the International Prospective Register of Systematic Reviews (PROSPERO), registration number CRD42024555048.

**Search strategy and information source**

The search strategy will be developed using the PICO (population, intervention, control and

outcomes) framework (33).

We will conduct an initial scoping search to identify relevant keywords and subject headings specific to each database and pilot test the search strategy in the following databases Medline, Scopus, and CINAHL to refine keywords and Boolean operators. The search strategy will finally be peer-reviewed by professional medical/academic librarians who are experts in systematic reviews to ensure comprehensiveness and accuracy in capturing relevant literature across the selected databases and sources. The search will be limited to studies conducted on humans and published in the English language. We plan to update the search during the process of writing the review manuscript to include new studies as they become available. Due to their comprehensive coverage of health, medicine, nursing, and psychological literature, searches will be conducted in the following databases: PsycInfo, Scopus, Web of Science, Medline, Cochrane, JBI EBP, CINAHL, Informit, and PubMed.

Each database will be searched for the concept of “Intimate partner violence” and “Breastfeeding” using a combination of subject headings and keywords.

We will manually search the reference lists of the retrieved articles to identify and include any additional relevant studies. The search strategies for the respective databases will be detailed in supplementary data (**S2 File**).

**Eligibility criteria**

**Inclusion criteria**

**Study design**

All peer-reviewed primary observational studies that specifically investigate the link between IPV and breastfeeding outcomes, such as early initiation, exclusivity, and duration among the perinatal population (1 year preconception, pregnant, 1 year post-partum) will be included. There will be no restrictions on the publication dates of the included studies.

**Exclusion criteria:**

- Non-peer reviewed articles such as; case reports and case series, editorials, letters to editors, commentaries, and opinion pieces without original research data, secondary or review articles, unless they contain primary data relevant to the study objectives.
- Studies focusing on populations other than the perinatal period (not including pregnant or postpartum women) or the general population.
- Studies focusing on IPV experiences of men.
- Studies that focused on specific population groups, such as individuals with chronic diseases like HIV or eating disorders.
- Studies that focus solely on individuals identifying as sexual minorities (e.g., transgender, homosexual individuals).

**Population**

We will include women who have experienced IPV and within the perinatal period who meet the following criteria:

1. Men as perpetrators of violence against women
2. Women who are in the postpartum period, with no contraindications to breastfeeding for either the mother or the baby
3. Women who are both victims of IPV and perpetrators of violence towards men

We will exclude the following women:

1. Mothers of multiple births (women who have delivered twins, triplets, or more)
2. Women with absolute contraindications for breastfeeding (e.g., galactosemia, maple syrup urine disease, phenylketonuria, or other metabolic disorders in the infant)
3. Mothers experiencing circumstances like stillbirth, neonatal death, or other situations where breastfeeding is not possible
4. Who are unable to breastfeed, for example, due to having undergone a mastectomy or with severe breast infections that significantly impede breastfeeding
5. Those who have eating disorders or chronic illnesses, such as HIV, and are taking specific medications that are harmful to the infant
6. Who have substance abuse issues, such as alcohol or drug use

**Concept**

**Intimate partner violence** is defined as any behavior within a current or former intimate relationship with a male that causes physical, sexual, or psychological harm, including acts of physical aggression, sexual coercion, psychological abuse, economic abuse (restricting access to financial resources, bank accounts or credit cards, education, employment and health care), controlling behaviour (restricting the victim's contact with family, friends, and social networks, constantly checking on the victim's whereabouts, communications, and activities, manipulation, regulating daily life) (2, 3). Physical violence includes intentional acts of physical force or aggression such as pushing, slapping, throwing, hair pulling, punching, kicking, or burning, use of a weapon, perpetrated with the potential to cause harm, injury, disability or death. Emotional/psychological violence is defined as acts or threats of acts, such as shouting, controlling, intimidating, humiliating, and threatening the victim. Sexual violence is defined as the use of force, coercion, or psychological intimidation to force a woman to engage in a sex act against her will whether or not it is completed.

Since IPV measurements or screening tools typically evaluate violence over the past year and lifetime (34), studies on perinatal intimate partner violence (P-IPV) vary in the timeframes they cover. To gather more comprehensive data demonstrating the risks of P-IPV, we define P-IPV in this paper as an abuse experience occurring 12 months before conception, throughout pregnancy, and up to 12 months after childbirth (35, 36).

The primary outcomes of this review and meta-analysis are the first three core Infant and Young Child Feeding (IYCF) indicators established by the World Health Organization (WHO): 1) the early initiation of breastfeeding, 2) exclusive breastfeeding (EBF) under 6 months, and 3) continued breastfeeding at least 2 years or beyond (37).

Early initiation of breastfeeding will be measured as the proportion of children born in the last 24 months who were put to the breast within one hour of birth. Exclusive breastfeeding will be measured as the proportion of infants 0–5 months of age who are fed exclusively with breast milk during the previous day and given no other foods or beverages except medication. Continued breastfeeding will be measured as the proportion of women who continue breastfeeding for up to 2 years or beyond.

**Context**

Studies conducted in any geographical location, with no restrictions on region or country, and including people of all ethnicities and races, will be included.

**Screening**

The retrieved studies from the search will be uploaded to Covidence (Veritas Health Information, Melbourne, Australia), an online software where duplicates will be identified and removed and the titles and abstracts, and full-text review will be independently screened by two reviewers (ZNA and CM) (38).

If any inclusion criteria are ambiguous based on the title or abstract, the study will be selected for full-text review. The full-text screening will be performed independently by two reviewers (ZNA and CM) using the same criteria. Any discrepancies during the screening process will be resolved by a third independent reviewer (LGS). The process of study inclusion will be illustrated using a flow diagram adapted from the PRISMA guidelines (39).

**Data extraction and management**

The data extraction plan for this systematic review and meta-analysis aims to systematically gather and accurately document relevant information from the included studies to investigate the impact of IPV on breastfeeding outcomes. Data extraction will be conducted using Covidence. Covidence provides customizable data extraction templates that ensure consistency and comprehensiveness in capturing relevant information from the included studies. This platform also facilitates collaboration among team members, allowing multiple reviewers to simultaneously work on data extraction while tracking changes and resolving discrepancies. The extracted data will be organized, filtered, and exported through Covidence for further analysis, ensuring a structured and consistent review process. Data extraction will be performed independently by two reviewers (ZNA and CM). Key information such as author(s), author affiliation, publication year, country of study, study design, funding sources, conflict of interest, ethics approval, sample size, participant demographics, IPV exposure details, adjustment variables, and breastfeeding outcomes will be recorded. Any discrepancies between reviewers will be resolved through discussion or decided by a third reviewer (LGS).

**Data analysis**

The extracted data will be analysed using STATA/ SETM Version 17. The analysis will begin with a descriptive summary of the included studies, detailing their key characteristics such as study design, population demographics, and outcomes. The pooled prevalence of IPV during the perinatal period will be calculated using a random-effects model.

We will assess heterogeneity among studies using the I² statistic and Chi-square test. To examine the association between P-IPV and breastfeeding outcomes, we will conduct meta-analyses to calculate pooled effect sizes. Given the anticipated variability among studies in terms of populations, settings, and methodologies, we will employ random-effects models.

To explore heterogeneity among the estimates of the primary studies, we plan to perform a subgroup analysis based on the study setting (region/s of the country where the study will be conducted), years of publication, sample size, and study population characteristics. Depending on the suitability of the data extracted, a meta-regression may also be performed to explore the potential sources of heterogeneity. Sensitivity analyses will ensure the robustness of our findings, and small study bias will be evaluated using funnel plots and Egger’s test. Asymmetric funnel plots will suggest potential publication bias. Depending on the data in the included studies, we will use odds ratios (OR) or risk ratios (RR), along with 95% confidence intervals (CI), to determine the association between P-IPV and breastfeeding outcomes. The results will be reported following the PRISMA guidelines.

**Risk of bias (Quality) assessment**

To ensure the rigor and reliability of this systematic review and meta-analysis, we will assess the risk of bias and the quality of the included studies. The risk of bias will be assessed using the Risk Of Bias In Non-randomized Studies - of Exposures (ROBINS-E) tool. ROBINS-E is a recent tool designed for systematic reviews to evaluate bias in observational studies. It includes seven key domains: confounding, participant selection, exposure classification, deviations from intended exposures, missing data, outcome measurement, and selection of reported results. Each domain is assessed through a set of signalling questions to gather crucial information about the study and its analysis (40). If randomized controlled trials (RCTs) are included, we will use the revised RoB 2 tool to assess the risk of bias. RoB 2 provides a framework for evaluating bias in a single estimate of intervention effects reported from RCTs (41).

We will conduct a thoughtful evaluation of each study's risk of bias categorizing them as low, moderate, and high. The Grading of Recommendations Assessment, Development, and Evaluation (GRADE) approach will be used for grading the overall quality of the body of evidence (42).

Two reviewers (ZNA and CM) will independently conduct the quality assessment and compare their results. Throughout the process, discrepancies between reviewers will be resolved through discussion and consensus. If consensus cannot be reached, a third reviewer (LGS) will be consulted to make a final decision.

**Discussion**

According to the WHO and United Nations Children’s Fund Global Breastfeeding Collective Scorecard (2018), less than half of newborns initiated breastfeeding within one hour of birth, and 41% were breastfed for up to six months (43). The target set by the Collective, which is led by UNICEF and WHO is to reach a global rate of 70% for early initiation of breastfeeding, 70% for exclusive breastfeeding, and 80% for continued breastfeeding to 2 years or more by 2030. To achieve such goals, interventions, and policies at various levels are needed to promote and support breastfeeding (44). Effective strategies include providing education and breastfeeding support before, during, and after delivery, involving not only mothers but also fathers, other family members, the health service/birthing attendant and, the broader community (45).

Breastfeeding can be influenced by a range of factors, including socioeconomic, cultural, biological, psychological, and obstetric service delivery factors (46-48). It has also been speculated that breastfeeding outcomes can be influenced by IPV. However, the literature on the subject is still scarce, and the study results are conflicting. Some studies indicate an association between IPV and breastfeeding, while others have not found statistically significant associations (49-52). However, many of these studies suffer from small sample sizes, poor study design, limited geographic and cultural diversity, lack of consideration for confounding factors, and heterogeneity in study populations. Currently, there are two systematic reviews examining the association between IPV and breastfeeding practices reviewing studies published between 2001 and 2019 (29, 30); this study expands and improves on these reviews. Collecting and synthesising evidence can be a step towards a better understanding of the impact of IPV on breastfeeding outcomes.

This review will synthesise the current body of evidence to explore and clarify the relationship between women’s experience of IPV during the perinatal period and breastfeeding outcomes. This systematic review and meta-analysis will update, compile, and critically review the evidence on the role of perinatal IPV on breastfeeding outcomes. The use of GRADE will result in an overall statement on the quality of the body of evidence for each outcome. In turn, this systematic review can then be used to inform future strategies and interventions for IPV in the perinatal period to support breastfeeding, thereby enhancing maternal and child health. This review has a limitation in that non-English language articles may be excluded, potentially increasing the risk of bias. However, to maintain transparency, we intend to exclude non-English language articles during the eligibility assessment stage.

**Ethics and dissemination**

Ethical approval is not required for this systematic review and meta-analysis, as it involves secondary analysis of published studies that are already available in scientific databases. The results of this review and meta-analysis will be submitted for peer-reviewed publication and presented at relevant scientific meetings, national and international conferences, and seminars.

**Updates to study protocol**

Should any modifications to the review protocol be necessary, these will be documented and included as supplementary information in the final manuscript and updated on the PROSPERO register.

**Supporting information**

**S1 File. Preferred Reporting Items for Systematic reviews and Meta-Analysis (PRISMA) checklist. (DOCX)**

**S2 File. Search strategies for included databases**

**(DOCX)**

**Acknowledgements**

We would like to acknowledge the academic librarians (Grai Calvey and Alexandra Martins dos Santos) for their assistance in drafting the search strategies and conducting comprehensive searches across various databases.

**Funding sources/sponsors**

No funding agency. This systematic review and meta-analysis will be conducted without funding from any government or research organization.

**Availability of data and materials**

The searches, studies, and data extracted for the review will be available upon reasonable request.

**Authors’ contributions**

ZNA conceptualized and ZNA, CM, and LGS developed the protocol, drafted and revised the manuscript, and provided critical feedback. ZNA holds primary responsibility for the final content. All authors have read and approved the final manuscript.

**Ethics approval and consent to participate**

Not applicable.

**Consent for publication**

Not applicable.

**Competing interests**

The authors declare no competing interests.

**Author affiliations**

^1^School of Health and Society, University of Wollongong, Northfelds Road, Wollongong, NSW 2522, Australia

^2^School of Public Health, University of Adelaide, Adelaide, SA, Australia

^3^Department of Women’s and Family Health, School of Midwifery, College of Medicine and Health Sciences, University of Gondar, Gondar, Ethiopia

**References**

1.	World Health Organization. Global and regional estimates of violence against women: prevalence and health effects of intimate partner violence and non-partner sexual violence: World Health Organization; 2013.

2.	Yount KM, Krause KH, VanderEnde KE. Economic coercion and partner violence against wives in Vietnam: a unified framework? J Interpers Violence. 2016;31(20):3307-31.

3.	World Health Organization. Violence against women prevalence estimates, 2018: global, regional and national prevalence estimates for intimate partner violence against women and global and regional prevalence estimates for non-partner sexual violence against women. World Health Organization; 2021 Mar 9.

4.	Devries KM, Kishor S, Johnson H, Stöckl H, Bacchus LJ, Garcia-Moreno C, et al. Intimate partner violence during pregnancy: analysis of prevalence data from 19 countries. Reproductive health matters. 2010;18(36):158-70.

5.	Marques DFdC. INTIMATE PARTNER VIOLENCE DURING PREGNANCY 2012 [Available from: [https://repositorioaberto.up.pt/bitstream/10216/72026/2/29312.pdf](https://repositorio-aberto.up.pt/bitstream/10216/72026/2/29312.pdf) [Access date; August 3, 2024 ]

6.	Taillieu TL, Brownridge DA. Violence against pregnant women: Prevalence, patterns, risk factors, theories, and directions for future research. Aggress Violent Behav. 2010;15(1):14-35.

7.	Saltzman LE, Johnson CH, Gilbert BC, Goodwin MM. Physical abuse around the time of pregnancy: an examination of prevalence and risk factors in 16 states. Matern Child Health J. 2003;7:31-43.

8.	Jasinski JL. Pregnancy and domestic violence: a review of the literature. Trauma, Violence, & Abuse. 2004;5(1):47-64.

9.	Charles P, Perreira KM. Intimate partner violence during pregnancy and 1-year post-partum. J Fam Violence. 2007;22:609-19.

10.	Martin SL, Harris-Britt A, Li Y, Moracco KE, Kupper LL, Campbell JC. Changes in intimate partner violence during pregnancy. J Fam Violence. 2004;19:201-10.

11.	Shanmugam D, Hou K, Pierson E. Quantifying disparities in intimate partner violence: a machine learning method to correct for underreporting. npj Women's Health. 2024;2(1):15.

12.	Schafer SD, Drach LL, Hedberg K, Kohn MA. Using diagnostic codes to screen for intimate partner violence in Oregon emergency departments and hospitals. Public health reports. 2008;123(5):628-35.

13.	World Health Organization. Intimate partner violence during pregnancy: Information sheet. 2011 [Available from: <https://iris.who.int/bitstream/handle/10665/70764/WHO_RHR_11.35_eng.pdf?sequence=1&isAllowed=y> [Access date; August 3, 2024]

14.	Alhusen JL, Ray E, Sharps P, Bullock L. Intimate partner violence during pregnancy: maternal and neonatal outcomes. Journal of women's health. 2015;24(1):100-6.

15.	Stöckl H, Filippi V, Watts C, Mbwambo JK. Induced abortion, pregnancy loss and intimate partner violence in Tanzania: a population based study. BMC Pregnancy Childbirth. 2012;12:1-8.

16.	Donovan B, Spracklen C, Schweizer M, Ryckman K, Saftlas A. Intimate partner violence during pregnancy and the risk for adverse infant outcomes: a systematic review and meta‐analysis. BJOG: An International Journal of Obstetrics & Gynaecology. 2016;123(8):1289-99.

17.	Shah PS, Shah J. Maternal exposure to domestic violence and pregnancy and birth outcomes: a systematic review and meta-analyses. Journal of women's health. 2010;19(11):2017-31.

18.	Stewart DE, Vigod SN, MacMillan HL, Chandra PS, Han A, Rondon MB, et al. Current reports on perinatal intimate partner violence. Current psychiatry reports. 2017;19:1-10.

19.	Campbell JC, Webster D, Koziol-McLain J, Block C, Campbell D, Curry MA, et al. Risk factors for femicide in abusive relationships: Results from a multisite case control study. Domestic Violence: Routledge; 2017. p. 135-43.

20.	UNICEF. Intimate Partner Violence and Breastfeeding; A Summary of the Evidence Base 2022 [Available from: https://www.unicef.org/media/128256/file/UNICEF_GBViE_Breastfeeding_Brief_September_2022.pdf [Access date; August 3, 2024]

21.	World Health Organization . Breastfeeding [Available from: <https://www.who.int/health-topics/breastfeeding#tab=tab_1>. [Access date; August 3,2024]

22.	Ogbo FA, Agho K, Ogeleka P, Woolfenden S, Page A, Eastwood J, et al. Infant feeding practices and diarrhoea in sub-Saharan African countries with high diarrhoea mortality. PLoS One. 2017;12(2):e0171792.

23.	Victora CG, Bahl R, Barros AJ, França GV, Horton S, Krasevec J, et al. Breastfeeding in the 21st century: epidemiology, mechanisms, and lifelong effect. The lancet. 2016;387(10017):475-90.

24.	Chowdhury R, Sinha B, Sankar MJ, Taneja S, Bhandari N, Rollins N, et al. Breastfeeding and maternal health outcomes: a systematic review and meta‐analysis. Acta paediatrica. 2015;104:96-113.

25.	World Health Organization. Improving Maternal, Newborn, Infant and Young Child Health and Nutrition. Essent. Nutr. Actions. Geneva: World Health Organization; 2013.

26.	Walters CN, Rakotomanana H, Komakech JJ, Stoecker BJ. Maternal experience of intimate partner violence is associated with suboptimal breastfeeding practices in Malawi, Tanzania, and Zambia: insights from a DHS analysis. Int Breastfeed J. 2021;16:1-12.

27.	UNICEF. Global breastfeeding scorecard, 2018: Enabling women to breastfeed through better policies and programmes. 2018.

28.	Tchakounte D, Kamga BF, Mbassa MRZ. The association between intimate partner violence and breastfeeding practices in Cameroon: An analysis from demographic and health survey of 2018. Heliyon. 2024.

29.	Normann AK, Bakiewicz A, Madsen FK, Khan KS, Rasch V, Linde DS. Intimate partner violence and breastfeeding: a systematic review. BMJ Open. 2020;10(10):e034153.

30.	Mezzavilla RdS, Ferreira MdF, Curioni CC, Lindsay AC, Hasselmann MH. Intimate partner violence and breastfeeding practices: a systematic review of observational studies. J Pediatr. 2018;94:226-37.

31.	Von Elm E, Altman DG, Egger M, Pocock SJ, Gøtzsche PC, Vandenbroucke JP. The Strengthening the Reporting of Observational Studies in Epidemiology (STROBE) statement: guidelines for reporting observational studies. The lancet. 2007;370(9596):1453-7.

32.	Moher D, Liberati A, Tetzlaff J, Altman DG, PRISMA Group* t. Preferred reporting items for systematic reviews and meta-analyses: the PRISMA statement. Annals of internal medicine. 2009;151(4):264-9.

33.	Schardt C, Adams MB, Owens T, Keitz S, Fontelo P. Utilization of the PICO framework to improve searching PubMed for clinical questions. BMC medical informatics and decision making. 2007;7:1-6.

34.	Wang Y, Fu Y, Ghazi P, Gao Q, Tian T, Kong F, et al. Prevalence of intimate partner violence against infertile women in low-income and middle-income countries: a systematic review and meta-analysis. The Lancet Global Health. 2022;10(6):e820-e30.

35.	Dai X, Chu X, Qi G, Yuan P, Zhou Y, Xiang H, et al. Worldwide perinatal intimate partner violence prevalence and risk factors for post-traumatic stress disorder in women: a systematic review and meta-analysis. Trauma, Violence, & Abuse. 2023:15248380231211950.

36.	Sharps PW, Laughon K, Giangrande SK. Intimate partner violence and the childbearing year: maternal and infant health consequences. Trauma, Violence, & Abuse. 2007;8(2):105-16.

37.	WHO U, USAID A, AED U. Indicators for assessing infant and young child feeding practices. Geneva: World Health Organization. 2008.

38.	Covidence. Covidence - better systematic review management.

Covidence;. 2024.

39.	Page MJ, McKenzie JE, Bossuyt PM, Boutron I, Hoffmann TC, Mulrow CD, et al. The PRISMA 2020 statement: an updated guideline for reporting systematic reviews. Bmj. 2021;372.

40.	Higgins JP, Morgan RL, Rooney AA, Taylor KW, Thayer KA, Silva RA, et al. A tool to assess risk of bias in non-randomized follow-up studies of exposure effects (ROBINS-E). Environment international. 2024;186:108602.

41.	Sterne JA, Savović J, Page MJ, Elbers RG, Blencowe NS, Boutron I, et al. RoB 2: a revised tool for assessing risk of bias in randomised trials. bmj. 2019;366.

42.	Guyatt GH, Oxman AD, Vist GE, Kunz R, Falck-Ytter Y, Alonso-Coello P, et al. GRADE: an emerging consensus on rating quality of evidence and strength of recommendations. Bmj. 2008;336(7650):924-6.

43.	Scorecard WGB. Enabling women to breastfeed through better policies and programmes. 2018. 2018.

44.	Masi AC, Stewart CJ. Role of breastfeeding in disease prevention. Microbial Biotechnology. 2024;17(7):e14520.

45.	Rollins N. Bhandari N, Hajeebhoy N, et al.¿ Why invest, and what it will take to improve breastfeeding practices. The Lancet. 2016;387(10017):491-504.

46.	Who U, Bank W. Levels and trends in child malnutrition. UNICEF/WHO/World Bank Group Joint Child Malnutrition Estimates. 2019.

47.	Semenic S, Loiselle C, Gottlieb L. Predictors of the duration of exclusive breastfeeding among first‐time mothers. Research in nursing & health. 2008;31(5):428-41.

48.	Child Y. Infant and young child feeding. Nutrition. 2011:11-3.

49.	Bullock LF, Libbus MK, Sable MR. Battering and breastfeeding in a WIC population. Canadian Journal of Nursing Research Archive. 2001.

50.	Silverman JG, Decker MR, Reed E, Raj A. Intimate partner violence around the time of pregnancy: association with breastfeeding behavior. Journal of women's health. 2006;15(8):934-40.

51.	Lau Y, Chan KS. Influence of intimate partner violence during pregnancy and early postpartum depressive symptoms on breastfeeding among Chinese women in Hong Kong. Journal of midwifery & women's health. 2007;52(2):e15-e20.

52.	Moraes CL, de Oliveira AS, Reichenheim ME, Lobato G. Severe physical violence between intimate partners during pregnancy: a risk factor for early cessation of exclusive breast-feeding. Public Health Nutr. 2011;14(12):2148-55.
