# Supplementary material for: Perinatal intimate partner violence and breastfeeding practices: A systematic review and meta-analysis protocol
Source: PLoS One. 2025 Feb 6;20(2):e0318585. doi: 10.1371/journal.pone.0318585 (PMC11801569; doi:10.1371/journal.pone.0318585)
Supplement: Attachment — Submitted filename: Review Report.docx [file pone.0318585.s004.docx]

Perinatal intimate partner violence and breastfeeding practices: a systematic review and meta-analysis protocol

Review Report

**Summary of Protocol:**

This systematic review and meta-analysis protocol aims to investigate the relationship between intimate partner violence (IPV) during the perinatal period and breastfeeding outcomes. The authors plan to examine early initiation, exclusive breastfeeding, and continued breastfeeding practices among women who have experienced IPV. The protocol outlines a comprehensive methodology including searches across nine major databases, rigorous screening and data extraction processes, and appropriate statistical analyses using STATA. The review will use ROBINS-E for risk of bias assessment and GRADE for evaluating the quality of evidence, with results reported following PRISMA guidelines.

Comments in short based on PLOS ONE guidelines -

1. **Strengths of the Proposal**:

The protocol addresses an important public health issue with clear objectives and well-defined methodology. It demonstrates strong technical rigor in its statistical approach and includes appropriate tools for quality assessment. The authors have carefully considered various aspects of the review process, from search strategy to data synthesis, and have properly registered the protocol with PROSPERO, showing good research practice.

2. **Comments and recommendations**:

As a reviewer, I find that this protocol is generally well-constructed. However, there are two key areas that warrant particular attention: (1) the theoretical framework or "theory of change" that explains the relationship between IPV and breastfeeding outcomes, and (2) the role of confounding factors within this framework.

***Theoretical Framework, Mechanisms and Literature Evidence:***

- Could you elaborate on the theoretical mechanisms explaining how IPV affects breastfeeding practices, particularly in fragile settings (Page 6)?
- It would be helpful to understand why this relationship might be of higher concern in fragile areas
- Perhaps you could incorporate some of the emerging literature (post-2020) to strengthen the background?
- Would it be possible to address how confounding factors not included in Backiewicz et al. (2020) might influence the IPV-breastfeeding relationship?
- Could you explain how this review will build upon previous systematic reviews, particularly regarding confounding factors that Backiewicz et al. did not consider?
- It might be valuable to include hypotheses about confounding relationships and their role in the theoretical framework

***Statistical and Methodological Considerations:***

- Have you considered implementing robust variance estimation for handling multiple effect sizes from the same study?
- Could you explain how this meta-analysis might overcome the data limitations that prevented Backiewicz et al. from conducting their meta-analysis?
- You might want to consider including plans for:
  - Combining different effect measures
  - Handling zero events and different follow-up periods
  - Treatment of adjusted vs. unadjusted estimates

***Inclusion/Exclusion Criteria and Study Design:***

- Could you provide additional rationale for some of the inclusion and exclusion criteria? For instance, what is the reasoning behind excluding mothers of twins? Would it be possible to include them as controls?
- The search strategy might benefit from additional terms for different types of IPV and breastfeeding measurement methods

***Data Management and Analysis***:

Some suggestions that might enhance this section include:

- Consider expanding the data extraction forms to incorporate measurement validation
- You might want to include a plan for handling missing data
- Perhaps add specific strategies for addressing publication bias
- Consider detailing your approach for exploring and handling heterogeneity
