# Supplementary material for: Perinatal intimate partner violence and breastfeeding practices: A systematic review and meta-analysis protocol
Source: PLoS One. 2025 Feb 6;20(2):e0318585. doi: 10.1371/journal.pone.0318585 (PMC11801569; doi:10.1371/journal.pone.0318585)
Supplement: Attachment — Submitted filename: Response to Reviewers.docx [file pone.0318585.s005.docx]

**Authors’ response to editor and reviewers’ comments**

**Editor comments**

1. First, the inclusion criteria do not seem clear or clearly justified: why are grey literature or preprints excluded? This seems undesirable given what we know about publication bias.

**Authors’ response:**

While we acknowledge the potential value of grey literature and preprints in preventing reporting bias, we prioritised academic databases in the search strategy for our systematic review and meta-analysis. Indeed, a global leader in evidence synthesis have published empirical evidence showing trials that are difficult to locate are often of lower methodological quality (1). This is because grey literature and preprints often lack formal peer review and are poorly reported. Including such articles could introduce greater variability, increase bias risk, and complicate data appraisal due to inconsistent reporting. This raises the concern that extensive literature searches, rather than preventing bias, might introduce it by including low methodological quality. Egger et al suggests that when resources are limited, prioritising thorough quality assessments is more critical than conducting exhaustive searches or translating articles (1).

2. I also found the criteria for the population unclear: what does it mean to refer to the "general population"?  What if there is an analysis that is a broader sample but includes many pregnant women and conducts analysis on this pregnant subsample; is this paper eligible and if not why not?

**Author’s response:** In our protocol, the term "general population" refers to studies that focus on women of reproductive age who are not specifically within the perinatal period (defined as one-year preconception, during pregnancy, and up to one year postpartum). These studies are excluded because our systematic review and meta-analysis aims to focus specifically on the unique relationship between women's experiences of IPV during the perinatal period and breastfeeding outcomes. For studies with a broader sample that includes many pregnant women and conducted analysis on this pregnant subsample, such papers would be eligible for inclusion. We have revised the description of the population on line number 258-259 to address this concern.

3. I also found the description of the subgroup analysis notably unclear: how will setting, years of publication, sample size and study population characteristics (what characteristics?) be used to define subgroups? Meta-regression seems more appropriate given what you (appear) to have in mind, but regardless, the analytical dimensions and the empirical strategy should be described in more detail.

**Author’s response:** We have revised the manuscript on lines 357-363 to include a more detail on subgroup analyses and introduced meta-regression. Briefly, the planned subgroup analyses aim to explore potential sources of heterogeneity in the included studies. Specifically:

- **Setting** will be used to define subgroups based on geographical regions (e.g. Europe, Sub-Saharan Africa), high-income versus low- and middle-income countries (as defined by the UN), or specific cultural contexts where the studies were conducted.
- **Years of publication** will be categorised into time periods, such as pre-2010 versus post-2010, to identify any temporal trends in IPV prevalence or breastfeeding practices.
- **Sample Size** will be used to group studies into categories based on the size of their study populations, such as small studies versus large studies, to examine whether sample size influences the outcomes.
- **Study Population characteristics** will include factors such as socioeconomic status, urban versus rural settings, and baseline health conditions, as reported in the included studies.

While subgroup analyses will help explore potential sources of heterogeneity, we agree that meta-regression may be more appropriate for examining continuous variables or interactions between factors. Meta-regression would allow us to quantitatively assess the influence of these variables and their contribution to heterogeneity. Thank you for this suggestion; we will consider using meta-regression to assess heterogeneity among the included studies.

**Reviewer 1 comments:**

1. Change the tense used in the paper to future tense

**Author’s response:** We respect the reviewer's feedback and acknowledge the comment. However, as noted by the Editor, the use of future tense is standard and appropriate for a protocol paper, as it reflects planned actions and methodologies. No changes were made to the manuscript in response to this comment.

**Reviewer 2 comments:**

**Summary of Protocol:**

This systematic review and meta-analysis protocol aims to investigate the relationship between intimate partner violence (IPV) during the perinatal period and breastfeeding outcomes. The authors plan to examine early initiation, exclusive breastfeeding, and continued breastfeeding practices among women who have experienced IPV. The protocol outlines a comprehensive methodology including searches across nine major databases, rigorous screening and data extraction processes, and appropriate statistical analyses using STATA. The review will use ROBINS-E for risk of bias assessment and GRADE for evaluating the quality of evidence, with results reported following PRISMA guidelines.

Comments in short based on PLOS ONE guidelines -

1. **Strengths of the Proposal:**

The protocol addresses an important public health issue with clear objectives and well-defined methodology. It demonstrates strong technical rigor in its statistical approach and includes appropriate tools for quality assessment. The authors have carefully considered various aspects of the review process, from search strategy to data synthesis, and have properly registered the protocol with PROSPERO, showing good research practice.

**Comments and recommendations**:

As a reviewer, I find that this protocol is generally well-constructed. However, there are two key areas that warrant particular attention: (1) the theoretical framework or "theory of change" that explains the relationship between IPV and breastfeeding outcomes, and (2) the role of confounding factors within this framework.

1. Could you elaborate on the theoretical mechanisms explaining how IPV affects breastfeeding practices, particularly in fragile settings (Page 6)?

**Author’s response:** The relationship between IPV and breastfeeding practices can be explained through a theoretical framework called the ‘deficit hypothesis’, which suggests that mothers experiencing violence face physical, psychosocial, or emotional barriers to optimal breastfeeding. The deficit hypothesis indicated that IPV impacts breastfeeding through several mechanisms, including physical effects such as sore nipples and difficulty with milk let-down, psychological impacts like depression, anxiety, and body negativity, and a lack of social or partner support. Additionally, controlling behaviours associated with IPV can reduce maternal autonomy, limiting access to healthcare and breastfeeding support services, while IPV-related mental health disorders further hinder breastfeeding practices (2). See the revised manuscript on lines 141-158

2. It would be helpful to understand why this relationship might be of higher concern in fragile areas.

**Author’s response:** In fragile settings, the challenges related to IPV and breastfeeding are intensified due to several interconnected factors. Firstly, these settings often experience higher rates of IPV, which can adversely affect breastfeeding practices. Secondly, the limited healthcare infrastructure and lack of support services for IPV survivors and new mothers make it challenging to address both IPV and breastfeeding-related issues effectively. Additionally, food insecurity, which is prevalent in fragile settings, is strongly associated with IPV and can directly hinder a mother's ability to breastfeed (3). Lastly, socioeconomic disadvantages, such as poverty, unemployment, and limited access to resources, further exacerbate the negative effects of IPV on breastfeeding outcomes (4, 5). See the revised manuscript on lines 150-158.

3. Perhaps you could incorporate some of the emerging literature (post-2020) to strengthen the background?

**Author’s response:** We have now included three of the recent literature published after 2020 in the revised manuscript.

4. Would it be possible to address how confounding factors not included in Backiewicz et al. (2020) might influence the IPV-breastfeeding relationship?

**Author’s response:** In their publication, Backiewicz et al wrote that there were 48 potential confounders identified from the 16 studies included in their review. However, Backiewicz et al did not list the confounders applied in each study, making it difficult for the reader to determine whether each individual study had made appropriate adjustments for confounding. If an important confounder was not included as an adjustment variable, then the IPV-breastfeeding association may be subject to residual confounding. Similarly, if a mediator has been inadvertently included as an adjustment variable, part of the total effect of IPV on breastfeeding will be ‘adjusted away’. We are applying modern epidemiological principles to the issue of confounding by preparing a Directed Acyclic Graph in advance of the review. This will help determine the potential for bias in each individual study.

5. Could you explain how this review will build upon previous systematic reviews, particularly regarding confounding factors that Backiewicz et al. did not consider?

**Author’s response:**

The review conducted by Backiewicz et al. has several limitations, outlined as follows:

1. While the review aimed to assess the association between IPV and breastfeeding outcomes, it included women exposed to other forms of violence (e.g., gang violence, bullying). Additionally, it included studies where IPV exposure occurred outside the perinatal period.
2. Women in intimate relationships lasting less than one month (during previous pregnancies, current pregnancy, or postpartum) were excluded without providing evidence to justify these exclusions.
3. The review did not explicitly list the confounders extracted from the included studies, making it unclear which specific confounders were excluded and how they might influence the IPV-breastfeeding relationship.
4. The Newcastle–Ottawa Scale (NOS) was used for quality assessment, which is nowadays considered a less rigorous tool. In contrast, we will use the ROBINS-E tool, a more robust and comprehensive quality assessment instrument.
5. The review did not perform a meta-analysis.
6. The scope of the review was not clearly defined. For example, the review included studies with IPV exposure occurring one year prior to pregnancy, during pregnancy, and postpartum, but it did not clarify the definition of the perinatal period. Furthermore, studies outside this timeframe were also included. In our systematic review and meta-analysis, we have clearly defined perinatal IPV as occurring one year preconception, during pregnancy, and up to one year postpartum.

So, this systematic review and meta-analysis will build upon previous reviews, particularly the work of Backiewicz et al., in several key ways.

First, we will expand the analysis of confounding factors by identifying additional confounders not previously considered and constructing Directed Acyclic Graphs (DAGs) to systematically explore how these factors influence the association between perinatal IPV and breastfeeding outcomes. Additionally, we will explicitly extract and analyse confounders from all included studies, providing a clearer understanding of their role in this relationship.

Second, we will enhance quality assessment by employing the ROBINS-E tool, which is more rigorous and advanced compared to the NOS used in previous reviews. This will ensure a more robust evaluation of study quality. We will also conduct sensitivity analyses based on study quality to further strengthen our findings.

Third, our review will focus on the timing of IPV exposure, clearly defining perinatal IPV as occurring within one year preconception, during pregnancy, and one year postpartum. This precise timeframe will allow for a more accurate assessment of IPV's impact on breastfeeding outcomes. We will also examine the timing of IPV exposure, distinguishing between IPV experienced before, during, and after pregnancy.

Fourth, we will conduct detailed subgroup analyses to investigate how different types of IPV; such as physical, sexual, and emotional—impact specific breastfeeding outcomes (early initiation, exclusivity, continuation). Additionally, we will perform meta-regression analyses to quantify the influence of specific confounders on effect sizes and identify sources of heterogeneity across studies.

Fifth, unlike previous reviews, our study will include a meta-analysis to provide quantitative estimates of the association between P-IPV and breastfeeding outcomes.

Sixth, we will apply stricter inclusion criteria, focusing exclusively on P-IPV and excluding other forms of violence. We will also avoid arbitrary restrictions, such as requiring a minimum relationship duration, which ensures a more targeted and valid analysis.

Lastly, we will incorporate the most recent studies published since Backiewicz et al.’s review, ensuring a comprehensive and up-to-date synthesis of the current evidence.

6. It might be valuable to include hypotheses about confounding relationships and their role in the theoretical framework

**Author’s response:** In our theoretical framework, we acknowledge that confounders such as maternal mental health, socioeconomic status, social support, and access to healthcare may influence both IPV and breastfeeding outcomes. These confounders will be accounted for in our analysis to isolate the direct effects of P-IPV. Hypotheses about their roles and pathways will be incorporated to strengthen the validity of our findings.

**Hypothesis**: Confounding factors, such as socioeconomic status, maternal age, education, parity, and cultural norms, influence both the risk of perinatal IPV and breastfeeding outcomes by shaping maternal stress, access to resources, and support systems. These confounders create indirect pathways that must be adjusted for to accurately assess the causal relationship between perinatal IPV and breastfeeding practices. See the revised manuscript on lines 206-2011.

**Statistical and Methodological Considerations:**

7. Have you considered implementing robust variance estimation for handling multiple effect sizes from the same study?

**Author’s response:** Yes, we will consider implementing robust variance estimation (RVE) to handle multiple effect sizes from the same study. We agree this is a valuable for addressing effect estimate multiplicity and dependency in meta-analyses (See line number 367-369 in the revised manuscript).

8. Could you explain how this meta-analysis might overcome the data limitations that prevented Backiewicz et al. from conducting their meta-analysis?

**Author’s response:** Thank you for raising this important question. We re-read the Backiewicz et al paper and note the limitations they indicated. They did not mention any specific *data*-related issues, but made recommendations for IPD, called for higher quality studies and better adjustment for pre-defined confounding. We have prepared a DAG in advance to define a minimum set of confounders and, where possible, we will combine in meta-analyses the studies with and without that minimum set. This will give us some indication of bias in poorly-adjusted analyses. We will contact authors for any data that is missing from publications that then allows us to include their study in our meta-analyses. Also, we have included some studies published since the Backiewicz et al. review which can potentially increase the number of high-quality studies available for meta-analysis (See the revised manuscript on lines 341-343).

.

9. You might want to consider including plans for:
   - Combining different effect measures
   - Handling zero events and different follow-up periods
   - Treatment of adjusted vs. unadjusted estimates

**Authors’ response:** We address each of your suggestions as follows:

1. Regarding **combining different effect measures**, we acknowledged the importance of this point and have included a detail plan on line 369-371. We specified our approach to standardising and pooling various effect estimates, such as odds ratios, risk ratios, and hazard ratios.
2. **Handling zero events and different follow-up periods is challenging.** We have added a section in our protocol (line 373-375) outlining our strategy for managing zero events, including the use of continuity corrections or alternative statistical methods. Additionally, we described our approach to addressing varying follow-up periods, such as utilising time-to-event data or standardising outcomes to a common time point where appropriate.
3. Regarding the **treatment of adjusted versus unadjusted estimates,** we recognised the importance of distinguishing between these types of estimates. We included a plan to handle them appropriately, such as conducting separate analyses or using meta-regression techniques to explore the impact of adjustment on our results. We revised our protocol to incorporate these data considerations on line 379-382.

***Inclusion/Exclusion Criteria and Study Design:***

10. Could you provide additional rationale for some of the inclusion and exclusion criteria? For instance, what is the reasoning behind excluding mothers of twins? Would it be possible to include them as controls?

**Author’s response:** The rationale for excluding mothers of twins in this systematic review and meta-analysis is based on several factors.

M**others of twins face unique breastfeeding challenges** that differ significantly from those faced by mothers of singletons such as increased demands on milk supply and the likelihood of medical complications.

Second, **breastfeeding patterns among twin mothers differ** from those of mothers of singletons. Twin mothers often have different rates of breastfeeding initiation, duration, and exclusivity, which could introduce greater variability to the analysis.

Finally**, limited data availability** is another consideration. Many studies on P-IPV and breastfeeding outcomes focus exclusively on singleton births. Including twins could significantly reduce the pool of available data for analysis, potentially limiting the strength and reliability of the meta-analysis.

With utmost respect, regarding the suggestion to include mothers of twins as controls, we are reluctant to adopt this suggestion. As breastfeeding patterns of twins differ to singletons, twins are not ‘inert’ and therefore any direct comparison of singletons against twin controls could distort the true relationship between IPV and BF. Instead, it would be more appropriate to conduct a separate analysis focusing specifically on the relationship between P-IPV and breastfeeding outcomes in mothers of twins, if sufficient data is available for future.

11. The search strategy might benefit from additional terms for different types of IPV and breastfeeding measurement methods

**Author’s response:** Our search strategy includes terms for various types of IPV, such as psychological, physical, and sexual violence, as well as economic abuse. Furthermore, we have incorporated a range of terms related to different breastfeeding measurement methods, including breastfeeding initiation, duration, exclusivity, and lactation practices (**Please see** **appendix, S2 File** for details**).**

***Data Management and Analysis***:

12. Some suggestions that might enhance this section include:

- Consider expanding the data extraction forms to incorporate measurement validation
- You might want to include a plan for handling missing data
- Perhaps add specific strategies for addressing publication bias
- Consider detailing your approach for exploring and handling heterogeneity

**Author’s response:** We have addressed all of these points in the revised protocol (lines 343-344, 356-365, 383-387).

1. We have expanded the data extraction forms to include measurement validation, ensuring that we capture reliable and standardised measurements from the included studies.
2. We have included a plan for handling missing data, outlining strategies such as imputation methods and sensitivity analyses to address any gaps in the data.
3. Specific strategies for addressing publication bias have been added, including the use of funnel plots and Egger’s test to assess the presence of bias in our included studies.
4. We have detailed our approach for exploring and handling heterogeneity, including conducting subgroup analyses and meta-regression to examine potential sources of variation across studies.

**References**

1.	Egger M, Juni P, Bartlett C, Holenstein F, Sterne J. How important are comprehensive literature searches and the assessment of trial quality in systematic reviews? Empirical study. Health technol assess. 2003;7(1):1-76.

2.	Kendall-Tackett KA. Violence against women and the perinatal period: the impact of lifetime violence and abuse on pregnancy, postpartum, and breastfeeding. Trauma, Violence, & Abuse. 2007;8(3):344-53.

3.	Ricks JL, Cochran SD, Arah OA, Williams JK, Seeman TE. Food insecurity and intimate partner violence against women: results from the California Women’s Health Survey. Public Health Nutr. 2016;19(5):914-23.

4.	Awungafac G, Mugamba S, Nalugoda F, Sjöland CF, Kigozi G, Rautiainen S, et al. Household food insecurity and its association with self-reported male perpetration of intimate partner violence: a survey of two districts in central and western Uganda. BMJ Open. 2021;11(3):e045427.

5.	Hatcher AM, Page S, Aletta van Eck L, Pearson I, Fielding-Miller R, Mazars C, et al. Systematic review of food insecurity and violence against women and girls: Mixed methods findings from low-and middle-income settings. PLOS global public health. 2022;2(9):e0000479.

6.	Mezzavilla RdS, Ferreira MdF, Curioni CC, Lindsay AC, Hasselmann MH. Intimate partner violence and breastfeeding practices: a systematic review of observational studies. J Pediatr. 2018;94(3):226-37.

**The End!!!**
